# Draft Genome Sequence of Violacein-Producing Marine Bacterium *Pseudoalteromonas* sp. 520P1

**DOI:** 10.1128/genomeA.01346-14

**Published:** 2014-12-24

**Authors:** Hoang Tran Dang, Kentarou Yotsumoto, Keiichi Enomoto

**Affiliations:** School of Environmental Science and Engineering, Kochi University of Technology, Kochi, Japan

## Abstract

Here, we report a draft 5.25-Mb genome sequence of *Pseudoalteromonas* sp. 520P1, a marine violacein-producing bacterium isolated from the Pacific coast of Japan. Genome annotation by BLAST searches revealed the presence of one acylhomoserine lactone (AHL) synthase (*luxI*) and five AHL receptor protein (*luxR*) gene homologs.

## GENOME ANNOUNCEMENT

*Pseudoalteromonas* sp. 520P1, a Gram-negative marine bacterium isolated from the Pacific coast of Japan, produces violacein only under static culture conditions (1, 2). Violacein, which is produced by several species of terrestrial and marine bacteria, is a secondary metabolite that possesses properties, such as antibacterial, antiviral, antitrypanosomal, and antitumor activities (3). These bioactive properties of violacein suggest that it could be used as a medicine or as a bio-dye because of its purple color. In a previous study, Wang et al. reported that the production of violacein by strain 520P1 was regulated by quorum-sensing mechanisms using an *N*-acylhomoserine lactone (AHL) (4). In *Vibrio fischeri*, two essential components in quorum-sensing-regulated bioluminescence, namely, AHL synthase (LuxI) and AHL receptor protein (LuxR), and their genes (*luxI*/*luxR*) have been revealed (5). However, homologous genes for *luxI* and *luxR* in strain 520P1 have not been reported so far. Identification of these genes is pivotal to understand regulatory mechanisms of quorum sensing and the nature of AHL(s) involved in violacein production. Therefore, we sequenced the whole genome of *Pseudoalteromonas* strain 520P1 no. 412 (NBRC 107704) to identify the *luxI* and *luxR* genes.

Genomic DNA of strain 520P1 No. 412 was purified using a QIAGEN Genomic DNA kit with a Genome-tip 100/G column (Qiagen KK, Tokyo, Japan). The genome was sequenced on an Illumina Hiseq 2000 system by Macrogen Japan (Tokyo, Japan). A total of 5,740,346 reads were assembled using SOAPdenovo Assembly into 67 scaffolds with an *N*_50_ length of 136,339 bp (6). The assembled draft genome sequence was approximately 5.25 Mb long with a total coverage of 110-fold and a G+C content of 34.96%. A total of 4,899 protein-coding regions and 99 RNA-coding sequences were detected using the GLIMMER system and RAST server, respectively (7, 8).

The draft genome was analyzed to locate quorum-sensing-related genes. Annotation and mapping using BLAST and the RAST server revealed that at least one *luxI* homolog and five *luxR* homologs were present in the genome of this strain. A pair of *luxI/luxR* genes was located on the same scaffold (scaffold 19) and the orientations of the *luxI* and *luxR* genes were in opposite directions.

Genomic analyses of two violacein-producing *Pseudoalteromonas* strains have been reported so far (9, 10). A comparative study of the genomes of these strains and strain 520P1 may help clarify the protein components involved in the quorum-sensing-regulation of violacein synthesis.

### Nucleotide sequence accession numbers.

This whole-genome shotgun project has been deposited in DDBJ/EMBL/GenBank under the accession no. BBIN00000000. The version described in this paper is the first version, BBIN01000000.
